# Feasibility of Point-of-Care Genomic Profiling in the Diagnosis and Treatment of Cancer of Unknown Primary

**DOI:** 10.1093/oncolo/oyad054

**Published:** 2023-03-18

**Authors:** Xin Wang, Andrea Beharry, Brandon S Sheffield, Parneet K Cheema

**Affiliations:** Medical Oncology Training Program, Department of Medicine, Temerty Faculty of Medicine, University of Toronto, Toronto, ON, Canada; Department of Laboratory Medicine, William Osler Health System, Brampton, ON, Canada; Department of Laboratory Medicine, William Osler Health System, Brampton, ON, Canada; Medical Oncology Training Program, Department of Medicine, Temerty Faculty of Medicine, University of Toronto, Toronto, ON, Canada; Division of Medical Oncology, Department of Medicine, William Osler Health System, Brampton, ON, Canada

**Keywords:** cancer of unknown primary, next-generation sequencing, molecular profiling, diagnostics, targeted treatment

## Abstract

**Introduction:**

Cancer of unknown primary remains a challenging clinical entity. Despite receiving empiric chemotherapy, median overall survival is approximately 6-12 months. Site-specific therapy based on molecular characterization has been shown to improve outcomes; however, feasibility outside of clinical trials, especially in community centers, is lacking. This study explores the application of rapid next-generation sequencing in defining cancer of unknown primary and to identify therapeutic biomarkers.

**Methods:**

A retrospective chart review was performed by identifying pathological samples designated cancer of unknown primary. Next-generation sequencing testing was based on an automated workflow utilizing the Genexus integrated sequencer, validated for clinical use. Genomic profiling was further integrated within a routine immunohistochemistry service, with results reported directly by anatomic pathologists.

**Results:**

Between October 2020 and October 2021, 578 solid tumor samples underwent genomic profiling. Among this cohort, 40 were selected based on an initial diagnosis of cancer of unknown primary. The median (range) age at diagnosis was 70 (42-85) and 23 (57%) were female. Genomic data were used to support a site-specific diagnosis in 6 patients (15%). Median turnaround time was 3 business days (IQR: 1-5). Most common alterations identified were *KRAS* (35%), *CDKN2A* (15%), *TP53* (15%), and *ERBB2* (12%). Actionable molecular targeted therapies were identified in 23 (57%) patients, including alterations in *BRAF, CDKN2A, ERBB2, FGFR2, IDH1*, and *KRAS.* Immunotherapy-sensitizing mismatch repair deficiency was identified in 1 patient.

**Conclusion:**

This study supports the adoption of rapid next-generation sequencing among patients with cancer of unknown primary. We also demonstrate the feasibility of integration of genomic profiling with diagnostic histopathology and immunohistochemistry in a community practice setting. Diagnostic algorithms incorporating genomic profiling to better define cancer of unknown primary should be considered for future study.

## Teaser

Cancer of unknown primary is a challenging clinical entity. This study explored the application of rapid next-generation sequencing to define cancer of unknown primary and identify therapeutic biomarkers.

**Implications for Practice**

Cancer of unknown primary is a challenging clinical entity both in terms of diagnosis and treatment. We demonstrate the feasibility of a rapid point-of-care molecular profiling test to be integrated as part of routine histopathology diagnostics. Furthermore, more than half of the patients had an actionable alteration identified, providing further evidence that molecular profiling should be part of the standard of care for patients diagnosed with cancer of unknown primary.

## Introduction

Cancer of unknown primary (CUP) comprises a heterogeneous group of metastatic malignancies. CUPs are defined when a histological metastatic cancer where a primary tumor cannot be identified after a comprehensive diagnostic workup including histopathology, immunohistochemistry, and radiological assessment.^1^ They represent approximately 2% of new cancers diagnosed each year.^2^ Patients can be divided into favorable and poor prognostic subgroups based on clinical features and whether localized treatments are possible.^1,3^ Despite multiagent cytotoxic chemotherapy, response rates to non-targeted therapy, even in modern cohorts, are generally poor in the range of 8-12 months.^4^

Biomarker-based prescription of anti-cancer therapy is the cornerstone of precision oncology. As the number of molecularly guided therapies continues to grow, the incorporation of genomic sequencing has the potential to identify actionable targets. Modern retrospective cohorts have identified clinically relevant genomic alterations in 85% of patients with CUP.^5^ Guidelines have emerged recommending ­panel-based next-generation sequencing (NGS) for patients with metastatic cancer and CUP.^1,6^ Furthermore, tumor agnostic biomarkers such as *NTRK* and microsatellite instability are already adopted as standard of care.^7,8^ Whether targeted therapy can outperform multiagent chemotherapy in patients with CUP is unknown and is being actively studied in the ongoing randomized phase II CUPISCO trial (NCT03498521).^9^

**Figure 1.**
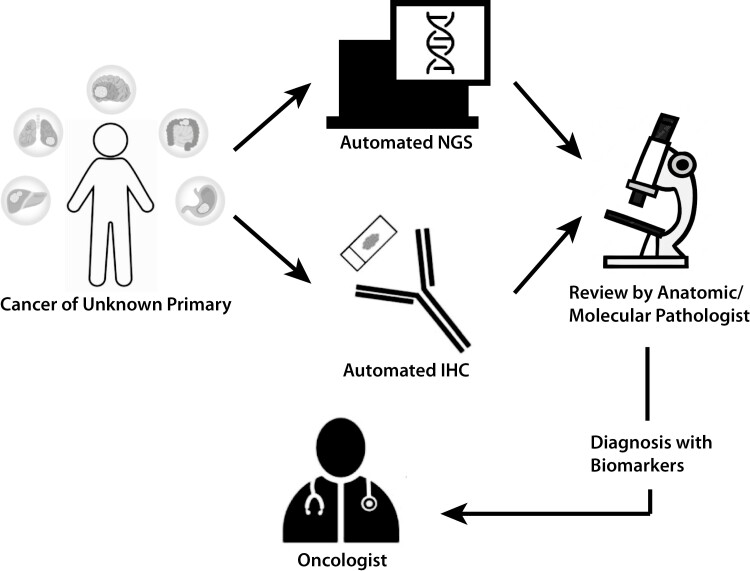
Proposed CUP diagnostic workflow. Anatomic pathologist simultaneously incorporates NGS results into a standard of care clinical history, imaging, and comprehensive immunohistochemistry.

Despite the promise of NGS, there remain significant barriers to access.^10^ These include lengthy turnaround times, upfront costs, and lack of availability in community-based oncology centers where the bulk of cancer patients are treated.^11^ Rapid NGS utilizes a high degree of automation, linking library preparation, gene sequencing, and bioinformatic analysis onto a single instrument.^12^ This allows comprehensive NGS to be performed in locations closer to patient care. Improvement in turnaround time has been shown to impact time-to-treatment as well as enrollment in clinical trials in patients with metastatic cancer.^13,14^ The purpose of this study was to evaluate the clinical feasibility of rapid NGS to guide the diagnosis and management of CUP patients in a real-world, community-based, oncology center (Fig. 1).

## Methods

### Study Population

This retrospective study was conducted for all patients undergoing clinical NGS between October 20, 2020 and October 12, 2021 at William Osler Health System, Canada, a large community hospital. Comprehensive review of patients’ medical records was performed to extract clinicodemographic details as well as pathology. Patients with an initial diagnosis of cancer of unknown primary were selected for detailed review using the electronic health record. This study was approved by the William Osler Health System research ethics board (REB#21-0028).

### Somatic NGS Testing

Formalin-fixed paraffin-embedded (FFPE) samples from biopsy samples were used. Tissue preparation, nucleic acid extraction, library preparation, sequencing, and bioinformatic analysis were conducted using the clinically validated Genexus integrated workflow that has been previously published.^11^ Ten nanograms of input DNA and RNA were used per sample. Assessment of tumor content and cellularity were performed by an anatomical pathologist prior to downstream testing. All NGS described in this study was performed using the Oncomine Precision Assay GX, an amplicon-based 50-gene panel including hotspot DNA analysis, copy-number assessment, and RNA fusion panel. These include hotspots such as *EGFR*, *ALK*, *BRAF*, *ROS1*, *RET*, *KRAS*, *PIK3CA*, and *ERBB2*. The panel includes 19 fusion variants as well as 14 CNV genes. Minimum depth of coverage at any amplicon is considered 500X to pass internal quality assurance. The assay is validated to detect SNVs and INDELs to an allelic fraction of 2.5%; however, lower frequency events can be called at the discretion of the treating pathologist.

### Evaluation of NGS Results

Clinically actionable alterations identified using the Genexus NGS workflow were classified according to the OncoKB annotations, which are stratified by level of evidence.^15^ Molecular results were interpreted and reported by the same anatomic pathologist, in conjunction with immunohistochemistry and morphologic findings, where applicable. Turnaround time was determined by diagnosis date to molecular report date for reflex (pathologist-initiated) testing, request date to molecular report date for bespoke (oncologist-initiated) testing, and accession date to molecular report date for referred-in testing. Turnaround time was measured as business days (excluding Saturdays, Sundays, and regional statutory holidays). Level of evidence based on OncoKB classification of FDA approved biomarkers.^15^

## Results

### Patient Characteristics

Between October 20, 2020 and October 12, 2021, 578 patient samples underwent clinical NGS testing, of which 40 were initially classified as CUP (Table 1). Among this subset of CUP patients, the median age was 70 (range 42-85), and 57% were female. Carcinoma not otherwise specified was the predominant histology (72%) with adenocarcinoma representing 17% of the cohort. Majority of patients had most of their tumor burden located above the diaphragm (65%). Median turnaround time for NGS from diagnosis to report in the medical record was 3 business days (IQR 1-5). The NGS failure rate was 0% in this cohort.

**Table 1.** Patient demographic and clinical characteristics.

Characteristic	NGS cohort (*n* = 40)
Age at sequencing, median (range)	70 (42-85)
Sex (%)	
Female	23 (57%)
Male	17 (43%)
Turnaround time, days (IQR)	3 (1-5)
Histology (%)	
Adenocarcinoma	7 (17%)
Carcinoma	29 (72%)
Other	4 (10%)
Location (%)	
Above diaphragm	26 (65%)
Below diaphragm	14 (35%)

### Site-Specific Diagnosis

In our CUP cohort, point-of-care NGS provided evidence to suggest a site-specific diagnosis in 15% (*n* = 6) of cases. Based on initial histopathology, 4 of the 6 cases had a differential diagnosis with a limited number of possible sites of origin (Supplementary Table S1). In 2 cases, the presence of *ERBB2* duplication prompted additional immunohistochemical analysis with mammaglobulin, GATA3, and GCDFP, which ultimately led to diagnosis of HER2 positive breast carcinoma. Case 384 had a differential diagnosis of pancreatobiliary versus upper GI primary. The presence of *IDH1 R132C* mutation was highly suggestive of intrahepatic cholangiocarcinoma and led to this patient enrolling in a clinical trial. In 2 cases (179 and 503) where initial pathology had features of spindle cell morphology, the presence of *CTNNB1* mutation suggested a diagnosis of desmoid fibromatosis, which is a benign entity. These 2 patients were subsequently enrolled under active surveillance. Finally, case 421 is a classic example of a patient with squamous cell features, where the identification of *NUTM1* fusion is pathognomonic for NUT carcinoma.

### Actionable Targets

Genomic drivers of non-CUP within our larger NGS cohort were previously published by Sheffield et al^11^ (Supplementary Table S2). Within the CUP cohort, most cases had at least 1 alteration identified, representing 80% (*n* = 32) of cases. One patient with no alterations identified had suboptimal DNA extraction. The most common alterations identified were *KRAS* (35%, *n* = 14), *CDKN2A* (15%, *n* = 8), *TP53* (15%, *n* = 6), and *ERBB2* (12%, *n* = 5). Actionable molecular targeted therapies were identified in 57% (*n* = 23) of patients (Table 2). Level 1 evidence based on FDA approved biomarkers predictive of response was present in 12% (*n* = 5) of patients. This includes 2 patients with MSI-high tumors with the indication for checkpoint immunotherapy, 2 patients with *ERBB2* amplification in the context of possible breast carcinoma, as well as 1 patient with *IDH1* mutation. Given FDA level of evidence is largely based on cancer subtypes, the majority of actionable biomarkers are off label indications, representing 61% of actionable alterations, encompassing 57% (*n* = 23) patients.

**Table 2.** Level of evidence for NGS detected therapeutic biomarkers.

Level of evidence^a^	% (*n*)^b^
Level 1	
* ERBB2*	5 (2)
* IDH1*	2 (1)
MSI-high	5 (2)
Level 4	
* BRAF*	2 (1)
* CDKN2A*	15 (6)
* FGFR3*	5 (2)
* KRAS G12C*	2 (1)
* KRAS non-G12C*	32 (13)
* PIK3CA*	5 (2)
None	42 (17)

^a^Level of evidence based on OncoKB FDA-approved drugs.

^b^Individual patients may harbor more than 1 actionable alteration.

## Discussion

This study aimed to explore the clinical feasibility of point-of-care NGS testing in patients with CUP in a real-world ­community-based setting. Access to NGS technologies remains a significant barrier for most patients, especially in community hospitals, where the majority of oncology care is provided. This is further hampered by significant delays in turnaround time as patient samples are routed to outside institutions.^16^ Our study is the first to demonstrate the diagnostic and predictive value of rapid NGS when integrated into the workflow of anatomic pathologists, as part of the evaluation for carcinomas of unknown primary.

Molecular features have been used to help clinicopathologic diagnosis in patients with CUP.^17^ Presence of certain driver mutations is known to be enriched in certain tumors. Furthermore, the addition of molecular information such as mutational signature may suggest etiological risk factors, that in addition to immunohistochemistry, may suggest a certain tissue of origin. Interestingly, 2 patients in our cohort with an initial suspicion of spindle cell malignancy, given the identification of *CTNNB1* mutation, were subsequently diagnosed with a benign tumor, and appropriately enrolled under active surveillance, possibly avoiding unnecessary chemotherapy-related toxicity. In our cohort, 15% of patients identified a genomic aberration that subsequently evoked an alternate diagnosis when evaluated in the context of clinical and pathologic review.

Two patients had actionable mismatch-repair deficiency, identified by immunohistochemistry. Additional patients had a site-specific diagnosis confirmed by immunohistochemistry based on findings from next-generation sequencing. In one such patient (case 423) with an alternate diagnosis of HER2+ breast cancer, they were then able to access standard of care palliative combination therapy with Paclitaxel, Trastuzumab, and Pertuzumab as per PERUSE study.^18^ While the importance of gene sequencing for this population cannot be understated, techniques such as histomorphology and immunohistochemistry remain critical. Here, the integration of NGS testing into an anatomical pathology practice highlights the strength of using multiple modalities to formulate a diagnosis and treatment recommendations.

Our findings, using a limited panel of genomic aberrations, are similar to other published modern CUP cohorts.^17,19^ In the Canadian treatment landscape, where funding for targeted therapies is directly tied to the cancer subtype, tissue of origin allows a clinician access to additional lines of approved therapy. Prior studies have shown that site-directed therapy had only a marginal benefit compared to empiric treatment.^20^ However, these studies largely predate the era of precision oncology and the growing number of commercially available molecularly targeted drugs, some of which are tumor agnostic.^21,22^

Modern CUP cohorts have shown that upwards of 20%-55% of patients may harbor actionable biomarkers based on OncoKB classification.^19,23^ Larger NGS panels, with their limited accessibility in public healthcare jurisdictions, have been used for comprehensive profiling of CUP patients. Using the FoundationOne assay, which includes 236 genes, 96% of cases had at least one alteration, but 20% potentially actionable.^5^ For the MSK impact assay, which includes 468 genes, 91% of patients had at least one alteration identified, and 30% included a potentially targetable alteration.^23^ Given the variability in assessing actionability, Varghese et al defined actionable using OncoKB resources and found 30% harbored a druggable alteration with FDA level 2-3 evidence. Even so, only 10% of them received a matched targeted therapy. This is similarly demonstrated in our cohort, with 80% (32 of 40) of patients having at least 1 alteration identified. With a rapid NGS panel, 57% of patients harbored a potentially actionable alteration. Among this group, 12% had a level 1 evidence biomarker in support of a molecularly targeted therapy. Despite this, the most common mutation identified in our cohort is *KRAS non-G12C* mutation, occurring in 32% of patients. Until recently, *KRAS non-G12C* remain undruggable. However, a RAF/MEK inhibitor has recently shown activity across a wide range of solid tumors and multiple myeloma.^24^ With the growing list of biomarker-based drug approvals, it is expected that genomics-informed therapies will play a significant role in patients with metastatic cancer.^1^

## Limitations

There are several limitations to our study. This was a retrospective analysis of a single-institution experience, although some patients were referred from other local institutions. Furthermore, the Oncomine Precision Assay interrogates a 50-gene panel, this clearly underestimates the full spectrum of genomic alterations in an attempt to facilitate rapid turnaround. Furthermore, small hotspot NGS panels are unable to elucidate certain important genome-wide biomarkers such as tumor mutational burden. Given the short follow-up, no survival and outcomes data were collected, which will need to be analyzed to demonstrate whether NGS used in the diagnostic workup of CUP can improve survival and therefore demonstrate clinical utility. Prospective trials comparing molecularly targeted agents to standard chemotherapy is being undertaken, such as the ongoing CUPISCO trial.^25^

## Conclusion

We report the feasibility of point-of-care biomarker testing using a panel-based NGS platform. This study demonstrates the feasibility and real-world application of this technology in a community-based oncology center. We propose a new diagnostic algorithm for CUP integrating rapid genomic profiling in the context of standard of care pathology incorporating clinical history, imaging, and immunohistochemistry (Fig. 1).

## Supplementary Material

oyad054_suppl_Supplementary_Table_S1

oyad054_suppl_Supplementary_Table_S2

## Data Availability

The data underlying this article will be shared on reasonable request to the corresponding author.
