# Supplementary material for: Feasibility of Point-of-Care Genomic Profiling in the Diagnosis and Treatment of Cancer of Unknown Primary
Source: Oncologist. 2023 Mar 18;28(6):474–8. doi: 10.1093/oncolo/oyad054 (PMC10243757; doi:10.1093/oncolo/oyad054)
Supplement: oyad054_suppl_Supplementary_Table_S1 [file oyad054_suppl_supplementary_table_s1.docx]

| **Patient Case #** | **Age** | **Sex** | **Biopsy Site** | **Initial Histopathology** | **Pathology Comments** | **Differential** | **Alterations** | **Interpretation** | **Final Primary Prediction** | **Treatment Implication** | **Reference** |
| --- | --- | --- | --- | --- | --- | --- | --- | --- | --- | --- | --- |
| 179 | 42 | F | Small bowel excision | Spindle cell lesion. Positive for KIT (CD117), negative for S100, desmin and CD34. | Possible GIST. | GIST | *CTNNB1 T41A* (c.121A>G). No alterations in *KIT* or *PDGFR* are identified | Given no immunoreactivity for DOG1, nuclear localization of beta-catenin, suggest fibromatosis rather GIST. Presence of CTNNB1 mutation highly prevalent in desmoid fibromatosis. | Desmoid fibromatosis | Active surveillance | Mod Pathol, 2012, Dec; 25(12): 1551-8 |
| 345 | 77 | F | Left axilla lymph node biopsy | Poorly differentiated carcinoma. Positive for AE1/AE3, CK7, and GATA3. Negative for ER, PR, SOX10, CK02, CDX2, NAPSIN, TTF1, E-cadherin, PAX8, p40, and melanoma markers (S100 and Mart-1). | Immunoprofile consistent with poorly differentiated carcinoma, but not site specific. | Breast vs Upper GI/Pancreatobiliary | *TP53 R175H, ERBB2 G778_P780dup* (activating exon 20 insertion in *HER2*), *ERBB2* copy ratio 2 | ERBB2 duplication led to additional staining, which were positive for mammaglobin, focal positive GFCDP-15, focal positive GATA3, suggesting breast carcinoma | HER2+ breast carcinoma | Paclitaxel for 1 cycle, developed TLS and transitioned to palliative care | Br J Cancer, 2004, May; 90(12): 2344–8, N Engl J Med, 2005, Oct; 353(16): 1652-4 |
| 384 | 62 | F | Liver nodule biopsy | Adenocarcinoma positive for CK7, negative staining of CK20, CDX2, PAX8, Synaptophysin, TTF1, p63, GATA3 focal positive. | Poorly differentiated adenocarcinoma with focal gland formation, possible primary include pancreatobiliary and upper GI | Pancreatobiliary vs Upper GI | *FGFR3 F384L, IDH1 R132C, FGFR3* loss copy ratio 0.67, MMR intact | Presence of IDH1 R132C alteration suggests intrahepatic cholangiocarcinoma | Intrahepatic cholangiocarcinoma | Matched to IDH1 clinical trial | Lancet Oncol, 2020, Jun; 21(6): 796-807 |
| 421 | 54 | M | Liver nodule biopsy | Carcinoma strongly positive CAM5.2 and p40, patchy positive AE1/3, p16 and EMA, diffusely weakly positive for TDT, rare cells positive for TTF1, CK7, CK20, CDX2, GATA3, NKX3.1, and CD45, and negative for chromogranin, synaptophysin, INSM1, Napsin, PAX8, CD5, CD117, Ber-EP4, SOX10, S100, and EBER. Ki-67 40%. RB is this RB1? and MMR intact. | Given keratin and p40, possible poorly differentiated squamous cell carcinoma | Squamous cell carcinoma | *NUTM1* fusion: *BRD4(14)-NUTM1(2)* fusion | NUTM1 fusion suggest midline NUT carcinoma | Metastatic midline NUT carcinoma | Palliative care due to poor PS | ﻿Am J Surg Pathol 2018;42:636–45, Mod Pathol, 2019, Feb; 32(6): 764-73 |
| 423 | 83 | F | Right lower lobe lung biopsy. | Needle biopsy shows poorly differentiated carcinoma given location suspicious for NSCLC, with diffuse positive CK7, negative for TTF1 and p40, no driver mutations identified (low expression of PDL1 (give %f PDL1 if we have it), negative EGFR can we reword this to no EGFR mutations by qPCR, negative by IHC: ALK, BRAF V600E, ROS1). | No actionable alterations identified, clinical correlation required to determine the primary of the tumor. | NSCLC vs Breast | *TP53 H179L*, *ERBB2* amplification 15 copy ratio | Given the finding of ERBB2 amplification, sample was stained for GATA3 and showed strong and diffuse staining, HER2 IHC 3+. | HER2+ breast carcinoma | Paclitaxel, Trastuzumab, Pertuzumab | Br J Cancer, 2004, May; 90(12): 2344–8, N Engl J Med, 2005, Oct; 353(16): 1652-4 |
| 503 | 48 | F | Left upper abdominal mass biopsy | Positive for cyclin D1. Negative for actin, desmin, CD34, CD117, S100, SMA, STAT6, keratin (AE1/AE3), and calretinin. | Sections show spindle cell lesion with prominent leloidal-type collagen and focal chronic inflammation. No epithelial elements. No significant mitotic activity, atypical mitoses or necrosis seen. DDx includes desmoid-type fibromatosis, GIST, low-grade sarcoma. | GIST vs Low grade sarcoma | *CTNNB1 pT41A,* c.121A>G mutation | Presence of CTNNB1 mutation highly prevalent in desmoid fibromatosis. | Desmoid fibromatosis | Low dose methotrexate with vinorelbine for recurrent unresectable disease | Mod Pathol, 2012, Dec; 25(12): 1551-8 |

Supplemental Table 1: Detailed clinical, immunohistochemical, and genomic alterations among CUP cases with NGS assisted diagnosis
